# The Stomach in Its Morbid States: Being a Practical Enquiry into the Nature and Treatment of Diseases of That Organ

**Published:** 1839-01

**Authors:** 


Art. V.

*The Stomach in its Morbid States: being a Practical Enquiry into the Nature and Treatment of Diseases of that Organ, and into the Influence they exercise upon the Origin, Progress, and Terminations of Diseases of the Liver, Heart, Lungs, and Brain.* By Langston Parker, m.r.c.s.—*London*, 1838. 8vo. pp. 303.

We hail this publication with pleasure, as evidently the production of a practical and at the same time a reflecting and experienced mind, and as one of a class of works in which medical literature has of late years been somewhat deficient. Zeal, industry, and talent have indeed abounded in the profession, but they have not succeeded in advancing the science of medicine in the same proportion as other sciences, or, perhaps, so much as might fairly have been expected from the ardour with which it has been cultivated.

One cause of this want of success is undoubtedly the complicated and difficult nature of the enquiry; but another, and we fear a very active one, is the limited and exclusive spirit in which medical investigations are too often carried on. From the multitude of phenomena, and the complexity of the relations which require to be observed, there is perhaps no science, for the successful cultivation of which extensive information, a comprehensive grasp of intellect, accuracy of observation, and caution in deduction are more imperatively required than in medicine. In the more elementary branches of knowledge, like chemistry, mathematics, or natural history, the facts are well defined, simple, and unchangeable; and what is once ascertained and placed in its natural order remains there for ever, unaffected by present theory or future discovery; and hence *they* may be mastered, and their boundaries extended, by persons whose general knowledge is not great, or whose minds are not remarkable for depth and comprehensiveness. But in medicine, and particularly in practical medicine, the facts on which we are to form our judgment and ground our treatment are of a very different kind. Instead of being definite and easily verified, the facts of medicine are living phenomena, or inferences from them, the true bearing of which is constantly liable to be affected by a multiplicity of active influences in and around us, and for the correct appreciation of which an ample stock of well-digested information and accurate reasoning powers are not less indispensable than well-disciplined powers of observation. Medicine being thus what may be called a *composite* science, requires for its successful cultivation not only a sufficient acquaintance with its constituent elements—anatomy, physiology, therapeutics, pathology, &c.,—but also the power and the habit of embracing in one point of view the inferences furnished by each; because, without the latter ground of security,—the correction of the one by the other,—the risk of being misled by partial facts is very great.

It has been from overlooking the true nature and aim of medical research, much more than from any want of talent in the profession, that while we have excellent anatomists, excellent physiologists, and excellent pathologists, who have each done much to advance their respective departments of knowledge, we have still comparatively few eminent *physicians*, or men who, skilled in all these elementary sciences, have excelled in applying their knowledge to the grand object of medical art, the prevention, relief, and cure of disease. If this opinion requires confirmation, we have only to go back for conclusive proof to the subject which has of late years most occupied the attention of the profession, *morbid anatomy*, and to observe the exclusiveness of spirit with which it has been studied. So intent have many of its cultivators been in investigating the mere changes of structure produced by or accompanying the progress of disease, that the causes which first gave rise to them, the signs by which they might be recognized during life, and the means by which their action might be arrested before actual disorganization ensued, have till of late been comparatively neglected by them. There are, it is true, some brilliant exceptions; but it must nevertheless be admitted that morbid anatomy has been studied by some of its most zealous cultivators as if its highest and ultimate object was, not the safety and recovery of the sick, but merely the accurate delineation, after death, of the organic ravages induced by disease during life. So true is this, that we could point out excellent pathologists to whose professional care we would not willingly intrust the treatment of a common fever or pleurisy; and we know more than one who would not willingly undertake such a charge.

If the above representation be at all correct, which in perfect sincerity we believe it to be, can we wonder that practical medicine has not advanced so rapidly as might have been expected from the progress of discovery in several of the more elementary sciences; or can we hesitate to believe that much more might be effected were the physician better disciplined in the use of his faculties, and taught to embrace a wider range in the observation of phenomena, and to consider his knowledge of them as complete only after having studied them in their various relations to each other and to the living whole?

These remarks were forcibly suggested to us by the attentive perusal, or we may say *study*, of Mr. Parker's book; and we are anxious to enforce them because convinced that they tend more directly to the extension of medical knowledge than if we had confined ourselves to comments on individual cases or propositions from its pages. In this opinion we are supported by no less an authority than that of Bacon, whose depth of penetration we every day see fresh cause to venerate and admire. In treating of the Advancement of Learning, he says "Let this ground be laid, that all works are overcome by amplitude of reward, *by soundness of direction*, and by the conjunction of labours. The first multiplieth endeavour, the second preventeth error, and the third supplieth the frailty of man; *but the principal of these is direction;* for *claudus in via antevertit cursorem extra viam;* and Solomon excellently setteth it down, 'If iron be not sharp, it requireth more strength; but wisdom is that which prevaileth:' signifying that the invention or election of the mean is more effectual than any inforcement or accumulation of endeavours. This I am induced to speak, for that, not derogating from the noble intention of any that have been deservers towards the state of learning, I do observe nevertheless that their words and acts are *rather works of magnificence and memory than of progression and proficience, and tend rather to augment the mass of learning in the multitude of learned men than to rectify or raise the sciences themselves.*" In strict accordance with this principle, we are anxious only for greater *soundness of direc-*

*tion* in medical investigations, in the full assurance that this, more than any mere augmentation of the mass of learning, is wanted to ensure the rapid rectification, "*progression, and proficience*" of the healing art. *False facts* are the bane of medicine more than of any other science; and we cannot but regard the partial knowledge which we refer to as the great source from which they spring.

In medical education, then, that branch ought to be considered the most important which combines and applies all the others to the elucidation of the nature, prevention, and treatment of disease; and yet this is precisely the part which has advanced the least, and to which least attention is still paid. It is true that clinical instruction has now a place in our curricula, and that it *ought* to do all this; but how rarely is it taught as it ought to be, and how little does it in reality interest the student! It *professes*, no doubt, to teach him to observe the outward expression of a disease, to investigate its less obvious signs, to distinguish it from others of an analogous nature, to devise proper principles of treatment, to choose his remedies appropriately, and to administer and modify them wisely and consistently; and, last of all, when death occurs, to trace the changes of structure which the disease has produced, and the relations which these bear to the symptoms observed during life: but where is such a rational and comprehensive course of instruction really given? When actually obtained, however, as it is now and then, the grand advantage of such a course is, that it disciplines the mind to patient and accurate observation and sound reasoning, and tends constantly to bring together into one useful whole the commonly disjointed branches of knowledge, which, in their separated state, serve only to "augment the mass of learning," and to mislead rather than to direct.

The work before us possesses many of the qualities which we are desirous of seeing combined in medical enquiries. Essentially practical in its character, it yet presents nothing of that exclusiveness of spirit which can embrace only one branch of a subject, and shuts out the consideration of all its general relations. Thus, while morbid anatomy is pursued by many as if the patient applied to the physician rather to ensure the honour of a *post-mortem* examination than in the hope of being cured, Mr. Parker seems to consider success in treatment as a primary object; and, while he attaches all due weight to the investigation of pathological changes, he attaches still more to the early recognition of the phenomena by which these changes are preceded; his primary object being that the existence of disease may be detected while it is yet curable, and the changes themselves, which no physician can undo, be effectually prevented. This, in truth, is the leading principle of Mr. Parker's investigations into the primary morbid states of the stomach, and we are not aware that he could have chosen a safer or more useful guide.

The subject of a work and its mode of treatment being determined, nothing tends more to clearness of exposition than simplicity of arrangement; for in books, as well as in speeches, it is a point of excellence to have a beginning, a middle, and an end; or, in other words, that things should follow each other in their natural order, and not be scattered about or unnecessarily intermixed. In this respect professional treatises in general cannot lay claim to much merit; and of the one more directly before us we are disposed to say that, although better than most others, it is still, in a practical point of view, susceptible of an improved arrangement. For example, when Mr. Parker so judiciously divided the primary morbid conditions of the stomach into three great classes,—the congestive or inflammatory, those characterized by lesions of sensibility, and those in which disordered secretions are the prominent phenomena,—he ought to have taken up each in succession, and exhausted all he had to say upon it before proceeding to another: but this he has not done. Beginning in the first chapter with "the morbid states of the stomach characterized by *increased vascularity*," he treats in the second of those "*dependent upon anæmia*," and in the third returns to a "general review of the *symptoms and sympathies* dependent on *vascular irritation of the stomach*." This is followed by one on its "confirmed inflammatory affections;" after which he treats of its nervous and secretory derangements: these constituting the substance of what we should call the first part of the book. In the second part, according to our view, although it is not so distinguished by the author, "the influence of the stomach upon other organs," and the influence of its diseases in exciting disease in them, are very ably discussed: after which the book ends with a chapter on Treatment, which abounds in sound sense and valuable practical observations.

In commenting upon this arrangement, we do not mean to deny that it possesses some advantages and affords facilities for consultation, but we cannot but consider it as faulty where our object is to acquire a comprehensive acquaintance with the morbid affections of the stomach. To say nothing of the impolicy of placing a chapter on *anæmic* affections in the midst of others treating of those of *increased vascularity*, we think that the seventh chapter, on the influence of the stomach upon other organs during health, ought properly to have been the first in the book, because a knowledge of the healthy sympathies of the stomach is required to understand the meaning of many of the symptoms attendant on its morbid states; and, had these been explained at the outset, *all* the cases would have been perused with increased advantage by the student. Further, we cannot help thinking that, by separating the subjects of the first, third, and fourth chapters so entirely, Mr. Parker has unnecessarily complicated his exposition, and subjected his inexperienced readers to the risk of believing the affections treated of to be distinct and separate diseases, rather than what they really are, different degrees of the same thing. We are quite aware that hyperæmia may exist without inflammation, but Mr. Parker himself admits that it is the first step towards it, and a simpler and more instructive arrangement would have been to adopt the *general* division of "morbid states of the stomach dependent upon congestion or inflammatory action," and to range hyperæmia, vascular irritation, and confirmed inflammatory action, as three subdivisions or successive degrees of the same thing. A greater unity in the detail of the symptoms and progress, in the investigation of the causes, and in the principles and *modus operandi* of the treatment, would thus have been ensured, and the risk been prevented of studying each as a separate entity dissimilar to the others; while at the same time considerable repetition would have been avoided.

There is still another point of view in which we think the work before us might, in common with many others, be greatly increased in value; and we allude to it now, not only from a conviction of its practical importance, but from believing that Mr. Parker will himself thank us for the suggestion. On reflecting on the most common difficulties of practice, every experienced physician will admit that the grand source of doubt and obscurity is, in most instances, the uncertainty in which we are left as to the true nature of the affection for which we are called upon to prescribe; and that, when we can obtain positive indications on that head, it becomes comparatively easy to determine what treatment ought to be adopted. In the case of stomachic affections, for example, as soon as we can ascertain clearly whether the symptoms depend on inflammatory action, on morbid sensibility, or on disordered secretions, the battle is half won; because we can then decide at once on the *kind* of treatment required, and have only to adapt it in degree to the individual case and constitution, to ensure the best results which its nature admits of. But when we meet with a case in which we cannot determine from what cause the symptoms arise, it is then we are puzzled how to act, and that errors in judgment, injurious to the patient and distressing to the practitioner, are apt to occur. In cases of this description, where the difficulty of a correct diagnosis is very great, it is rather from a careful consideration of the peculiarities of constitution, the nature of the exciting causes, and a minute history of the disease, than from the prominent symptoms, that we draw our conclusions and decide on the means of cure. In all cases, indeed, whether obscure or not, an accurate acquaintance with these modifying circumstances is of great use in planning and conducting the treatment, and protecting the patient against future attacks, and therefore they ought always to be noticed. Without them the narrative of a case is incomplete and comparatively uninstructive; and, as a few lines suffice for the purpose, there can be no good excuse for omitting them.

To apply these remarks to Mr. Parker's work, we have only to bear in mind the difficulty which is frequently experienced in deciding whether digestive derangement arises from inflammatory action or from morbid sensibility, and the importance of everything from which assistance can be obtained in arriving at the truth. Mr. Parker mentions several patients, for example in whom great epigastric pain, aggravated on pressure, strong pulsations, fulness, and all the usual symptoms of inflammatory excitement, were produced by the opposite morbid state, and consequently aggravated by leeches and low diet, while they were cured by wine, nourishing food, and tonics. Similar occurrences are by no means rare with other organs as well as the stomach. Thus, great excitement of the brain arises from an exhausted as well as over-stimulated state of the system, and is then appeased by food and wine, which in the opposite state would be destructive of life. This is seen even in the delirium of fever, which in the commencement of the disease is generally caused by excess, and in the latter stages more frequently by deficiency of stimulus. In all such cases, then, the safety of the patient depends wholly on the accuracy of our decision; and yet, where the prominent symptoms are thus alike, it is only from a consideration of the modifying causes above noticed that we can select any grounds for an opinion.

Influenced by considerations of this kind, we have long thought (and our opinion is confirmed by the perusal of Mr. Parker's cases and remarks,) that the constitution, previous history, and habits of the patient, and the causes of disease, are not generally enquired into with sufficient minuteness; and that much valuable information, applicable to both the diagnosis and treatment, is thus lost; while the patient is, on his recovery, left to run the same course, unwarned of the risk to which it exposes him, till he finds himself becoming the prey of another attack. That an acquaintance with the individual constitution, taken along with other considerations, affords an additional clue to the nature of the morbid state, will scarcely be denied by any reflecting practitioner. Thus, we find that a person of a full and strong habit of body is prone to affections of an inflammatory character; while another, of a highly excitable nervous temperament, will be predisposed rather to affections attended with increased sensibility without vascular irritation. From this peculiarity, the same cause may produce a different form of disease in each. Where, for example, indigestion is brought on by intemperate eating, (by which we mean eating more than the system and mode of life require,) in a constitution of low excitability, the affection will most probably be at first, and continue for a shorter or longer time, of a purely congestive character, leading gradually to glandular obstructions and morbid secretions; while the same cause operating in a person of a sanguine temperament and excitable circulation, will be more likely to induce inflammatory action, and all its attendant consequences. If, again, the constitution be highly nervous and irritable, and the cause be anxiety of mind, the sensibility of the stomach may become exalted without real vascular excitement, and the most prominent symptoms be those arising from the sympathetic disturbance of other organs. But as, from a variety of circumstances, each of these states is constantly liable to be converted into one of a different kind, it is obvious that, although we may derive much assistance in forming a correct diagnosis from considering the relation between the exciting cause and the constitutional tendency, we ought never to trust to the latter alone, but to examine *all* the phenomena, and the changes which occur in them, with scrupulous care, before finally determining upon our course of action.

An acquaintance with the *causes* as well as the constitution is not less useful in throwing light upon the nature of disease, and affording indications for its successful treatment; and we are therefore desirous that, in a narrative of cases, they also should be more distinctly stated. It is true that they are often difficult of detection, but we have ever found it an excellent rule in practice to take it for granted that an active cause *does* exist, whether we can find it out or not, and consequently never to lose sight of any opportunity of getting at it which a better acquaintance with the patient's condition or private history may afford. It is astonishing how often and how completely we may be misled if we trust implicitly to the statement of the patient, especially in answer to a general question, where a different meaning may be attached to the same words. Thus, when we enquire whether he is aware of any cause for his ailments, he will necessarily answer according to what he believes to be causes, and may confidently, and honestly, and rationally reply in the negative, where, on a little examination, a cause, very palpable to a practitioner, becomes immediately apparent. About a year ago, for example, we were consulted for severe indigestion, by a strong, healthy-looking man, in the prime of life, who declared that he knew no cause whatever for his ailments. On our expressing an opinion that a very powerful cause *must* be in operation to induce such a state in a person of his formation, and catechising him closely as to his habits, it came out that he was extremely fond of shooting and athletic sports; and that in the highlands, where he chiefly lived, he was in the custom of walking out after a good breakfast, and spending the whole day on the hills, without further support, till he returned nearly exhausted to a six-o'clock dinner, to which he sat down famishing, and, after eating almost to oppression, he fell into a deep sleep, from which he awoke disturbed by dreams and flatulence. So far from considering this mode of life as the cause of his indigestion, his wonder was why he, who was so constantly in the open air, and took so much exercise, should nevertheless have an inefficient stomach! In another instance we were lately assured by a lady, to whom we were called in the country, and where we had not an opportunity of seeing the evacuations, that her bowels were in perfectly good order and acted daily; when, on examining the abdomen to remove our scepticism on that point, we found it distended by fæces. On ordering a succession of active purgatives and injections for their removal, the patient remonstrated, and stated that, as she had repeatedly of late taken salts, which operated freely, it would weaken her too much to give her more, and that she could not require them. We of course insisted; and when, to her great surprise and relief, an enormous quantity of solid fæces made its appearance, she then said that it was *many weeks* since she had passed a natural evacuation *of that kind;* but that, not knowing that liquid excretions were insufficient, she had answered our questions as she did. In a third instance, of very recent occurrence, we were assured by a gentleman of much good sense that he knew no cause for a continual irritation subsisting in the stomach and bowels, attended by great depression of spirits and sleeplessness, and that all his habits were regular and temperate. It turned out, however, that, feeling much debility, he was eating meat twice a day, by way of keeping up his strength; making a very long fast between breakfast and dinner, on account of business; taking a glass or two of wine after dinner, *to relieve exhaustion,* and a good supper and spirits and water at night, *to promote sleep;* and, to crown all, taking frequent irritating purgatives, *to carry off the offensive matter* which lodged in his bowels and caused the depression! As he had an apparently good reason to assign for each of his practices, and felt immediate relief from his purgative, it never occurred to him that any of them could be looked upon as the real *causes* of his sufferings; and, even when the fact was pointed out to him, he was at first not a little incredulous. The excess of food, the long fast, the supper, the spirits, and the purgatives were evidently the original causes of the irritation; and, when these were obviated, and the diet for a time restricted to mild farinaceous food, without wine, he began to recover: still, however, occasional relapses arose, chiefly from the abuse of purgatives, which he was unwilling to give up, because, as he said, he *felt* relief from them, and they must therefore do good. Ultimately his eyes were opened to their true effects also, and he got well much sooner than he expected.

We have insisted, at some length, upon the necessity of careful examination into the facts of the case, because it is peculiarly in stomach affections that both patient and practitioner are apt to be deceived, unless the latter satisfies himself by his own observation, and that a right regimen (using that term in its widest sense) can accomplish more than is commonly imagined. If the practitioner contents himself with merely ascertaining what the affection is and prescribing medical treatment for its relief, he leaves the half of his duty undone, and the patient speedily relapses into worse than his former state. In this respect we cannot but consider the majority of Mr. Parker's cases as defective. He rarely mentions the natural constitution of his patients, their past habits and mode of life, or the numerous and important conditions of *general* regimen, which are at least as essential to recovery as the purely medical part of the treatment. The consequence is, that his cases are not so instructive to others as a few particulars of this kind would have made them; and that the opportunity is lost of connecting the appearance of the disease with the errors of regimen which produced it, and consequently the clue which they offer to a proper curative and preservative treatment is not sufficiently appreciated. Mr. Parker, indeed, is not the only author against whom this charge may be brought. The defect of which we speak is common; and, as it has an injurious influence on practice, we shall offer an example or two in illustration of our meaning.

On opening the volume, the first case which presents itself is one, p. 68, entitled "morbid sensibility of the stomach with epigastric pulsation." It is there stated that "a lady labouring under hepatic disease became affected, during its progress, with pain after taking food, occasional vomiting, and tenderness in the epigastrium of a slight character. There was strong pulsation at all times visible to the eye, and communicating a powerful impulse to the hand laid upon it. Her dyspeptic symptoms and the pulsation disappeared after one or two applications of leeches, succeeded by blisters."

Now we submit that such a narrative conveys the minimum of information which any case can afford, and that its whole meaning is made unnecessarily to rest on our estimate of Mr. Parker's skill and trustworthiness, (of both of which, however, we have a high opinion;) whereas ten lines more might have made it replete with useful instruction and stable on its own foundation. The cause, its relation to the peculiarities of constitution, its mode of operation, the nature of the disease, and the rationale of the cure, are all left to conjecture; and, indeed, from the relief of the symptoms by leeches and blistering, we are led to imagine that the case was one of inflammatory congestion, rather than of morbid sensibility. But how much more instructive would it appear, were it to be mentioned that, 'after exposure to mental anxiety, a lady of a given age, of a *nervous and irritable constitution and previously delicate health, accustomed to a sedentary mode of life and full diet*, became affected with hepatic disease, during which she suffered from pain after taking food, occasional vomiting, tenderness in the epigastrium accompanied by strong pulsation visible to the eye; and that, by the application of a few leeches, followed by quiet of mind, mild diet, and gentle exercise, she speedily recovered.' We know nothing of the real history of the lady; but, supposing it to be as we have stated, all the parts of the case immediately throw light upon each other, and the rationale of the treatment becomes so self-evident, that no reflecting mind can read the statement without carrying away from it definite principles of action applicable to other cases, and applicable with such modifications as a difference in the elements may require. This is, in our opinion, the only or chief value of cases, and we suspect that the very different results obtained by different practitioners from the use of the same remedies is to be ascribed to the neglect of these more important particulars. We admit most readily that all Mr. Parker's cases are far from being so meager; on the contrary, many of them, as well as the remarks which follow them, are very instructive; but they might *all* be greatly increased in practical value and present interest by the addition of a few sentences explanatory of the conditions which we have mentioned.

That this constant reference to the habits and constitution of the patient is of real importance in practice will be still further evident from a survey of the cases above narrated, as having lately occurred to ourselves. Had we implicitly relied on the first patient's assertion, that there was no cause for his indigestion, we might have sent him back to the mountains to remain a sufferer, because he would have returned unsuspectingly to the mode of life which excited the evil. Not aware of the necessity of renewed supplies of nourishment to the system at proper intervals, he made long fasts even while using great bodily exertion; and not aware that bodily fatigue *exhausts the stomach* in common with other organs, he sat down to his evening meal on his return home before the fatigue went off; and, believing that his day's exertions required an ample dinner, he ate almost to repletion; and, overcome by fatigue and the distension of stomach, fell forthwith into a profound sleep, which still further impaired the digestive powers. Here, it is obvious, no permanent cure could be effected without a direct reference to the past history and exciting cause. On various occasions, accordingly, he had come to town for advice; and, by local depletion, moderate diet, and mild laxatives, speedily obtained relief; but, invariably on his return to the country, the symptoms recurred, much to his own surprise, as he always understood that country air and exercise were the best cures for indigestion. On explaining to him the errors into which he had fallen, and urging him to adapt his mode of life more closely to the laws of his constitution, he had no difficulty in understanding the causes of his relapses, and promised to avoid them in future. When we last heard of him, he was much better, but not entirely well.

In the third case, again, the patient, who was a man of much good sense, conceived himself acting very properly when he took much nourishing food and wine, to relieve the sense of debility; spirits and water to procure sleep; and constant purgatives, to relieve the depression and carry off offensive matter. From all of these practices he felt *immediate* relief; and thence, naturally inferring that they were beneficial, he had no hesitation in declaring that he knew no cause whatever for his ailments: and yet, to any one who was acquainted with the human functions, it was manifest that he was taking twice the quantity of nourishment which his weakened stomach could digest or his sedentary mode of life require; that the wine merely relieved the oppression caused by the undigested food, and that the supper and spirituous drinks merely gave him rest by nightly inducing a state of oppression akin to apoplexy. In his former treatment most of these things were overlooked; and hence, after having been twice relieved by physicians under whose care he placed himself at a distance, he also relapsed on his return. But when his reason became convinced, he changed his whole system of living, and with the best results; for, when we saw him lately, he presented a healthy clearness of countenance and alacrity of mind to which he had been long a stranger.

We have already mentioned, as a strong recommendation of Mr. Parker's work, that the author is not *exclusive* in his views, but endeavours to see and represent things as they really exist in nature, and to rectify his own observations by the experience of others. In confirmation of this remark, we would refer to the discrimination exhibited in almost every chapter of the book, and to the whole tenor of his remarks on the morbid states of the stomach characterized by increased vascular action. So far from resting satisfied with a single examination of a case, Mr. Parker inculcates the necessity of a watchful observance of its progress and changes throughout its whole course, and shows, for example, that an affection, which is undoubtedly inflammatory at its commencement, sometimes passes into the opposite condition in a very short time, the original symptoms remaining so essentially the same as to mislead the incautious observer into the belief that the inflammatory character continues. Leeching and low diet may thus be very appropriate remedies at the outset, where their repetition and continuance—apparently warranted by their first good effects—really aggravate all the symptoms, and thereby seem, to those who do not recognize the actual change in the morbid state, to call for still further depletion. Occasionally, indeed, the assemblage of symptoms occurring in the one state is so identical with that usually observed in the other, that it is chiefly by attention to the constitution and previous history of the patient that we can arrive at an accurate diagnosis. Mr. Parker mentions a case of this kind which exhibited, "*in the most marked degree*, all the symptoms attendant upon the incipient stages of inflammatory disease of the stomach." "The pain after food, tenderness in the epigastrium on pressure, vomiting, scanty urine," (p. 15,) which commonly denote an inflammatory state, were all present, and yet it was in reality a case requiring the use of tonics, porter, and animal food; and under their influence speedy recovery ensued.

The same discriminating comprehensiveness of mind is conspicuous in Mr. Parker's remarks on the indications afforded by the state of the *tongue*, and their frequently deceptive character; and also in what he says on the necessity of a careful examination of the epigastric and hypochondriac regions, and the utility of *percussion* in determining the real nature of the gastric affection. The observations on *appetite* and on *nausea* are also very good. According to Mr. Parker, the appetite is rarely defective so long as the inflammatory irritation of the mucous coat is limited in extent, and it is null only where the disease occupies the whole or a large portion of the mucous surface. *Nausea* he considers an almost invariable attendant on inflammatory indigestion, and it occurs either immediately or within two or three hours after taking food. In another place he notices the supervention of *acute pains* in the chest resembling pleurisy, which, being accompanied by *cough* and *quick*

*breathing*, are often ascribed to pulmonary disease, and consequently mismanaged; when, in fact, they are caused entirely by chronic gastritis. Many persons die, he says, of affections thus induced, who might have been easily cured if the true nature of the disease had been detected at the beginning. This is peculiarly apt to happen in children, in whom "hurried breathing is often the most marked symptom of gastritis," when occurring along with occasional sickness and crying. The stethoscope and direct auscultation are here precious resources, from the negative evidence which they afford. Of *vomiting*, as a symptom of gastritic inflammation, he thinks with Andral, that it occurs principally "where a chronic state assumes the acute type, or where an obstacle is offered to the free passage of the food either in its entrance to, or exit from, the stomach." (p. 43.) It is far from being an attendant on all cases of gastritis.

On *morbid sensibility*, as a cause of indigestion, there are many valuable observations which we cannot stop to notice; and here, as elsewhere, the author avails himself of the experience of other writers to perfect his own views. In this section of the book, are to be found two or three excellent cases; and, at p. 58-9, some interesting comments occur on the occasional combination of the inflammatory with the nervous form of disease—a peculiarity which increases the difficulty of treatment, and which it requires all the care and sagacity of the physician to meet successfully.

The chapter on "the morbid states of the stomach, characterized by *disordered secretions*," is of an equally practical character with the rest, and is also illustrated by some apposite cases. One of these, which is particularly instructive in its details, we shall here give at length.

"A lady, aged twenty-nine, one year married, had suffered for three months prior to her consulting me from total loss of appetite, weight and uneasiness in the epigastrium, constant nausea, with frequent vomiting of food, and mucous discharges. She had constant headach, seated in the forehead and temples, and a total indisposition to sleep. The breathing was quickened, and there was a short, dry, frequent cough. The lungs, on examination, afforded no evidence of disease. Leeches, blisters, and counter-irritants, had been applied to the epigastrium, with the effect rather of aggravating than relieving any of the symptoms of stomach disorder. Aperients, in various forms, had been tried to their fullest extent; opiates had also been administered, with a view of procuring some rest: but all had failed. The patient actually passed night after night without closing her eyes. Leeches were now applied to the temples and behind the ears, and these were succeeded by blisters: these remedies aggravated the pain in the head, and brought on delirium. The stomach still continued in the same state, in spite of all that had been done; whilst the sympathetic irritation of the head and chest were worse. I now reflected whether I had not to treat one of those diseases which depend upon the accumulation of a mass of morbid secretions in the stomach and first passages, and which, though admitted as a fruitful source of disease by Stoll, Tissot, Finke, Pomme, De Larroque, and others, had been almost ridiculed out of the domain of pathology by the doctrine of irritation substituted by Broussais, in which all these secretory disorders are considered as the result of inflammatory action. Finding that local depletion, blisters, counter-irritants, and aperients had failed, whilst the state of the patient, the heat of skin, and arterial excitement, completely prohibited the exhibition of tonics, I determined to try the effects of the tartar emetic, from which vast benefit had been derived in such instances, both by De Larroque and Andral. Two grains of this substance were administered. The patient vomited an immense quantity of bilious and mucous fluids; the stomach became comfortable, the sickness and nausea disappeared, and for two days the head remained free from pain. In a few days the symptoms returned in a milder form. The remedy was again employed: vomiting of the same discharges to a much less extent. After a third repetition there was no more return of complaint; the patient became perfectly convalescent. *Remarks.*—This case exhibits two or three points which it is important to notice:—1st. That secretory irritations of this kind may continue for an indefinite period without being accompanied with any real inflammatory action of the stomach. 2dly. The sympathetic affections which they excite are sometimes of a purely nervous character and aggravated by all antiphlogistic treatment, as the present case shows. We need only refer to the effects of treatment to establish this." (p. 83.)

The most important division, however, (or rather what ought to have been a division,) of Mr. Parker's treatise, and perhaps the one which he has treated most ably, is that in which he discusses the influence of morbid states of the stomach on the origin and progress of disease in remoter organs. In one sense, indeed, it cannot be affirmed that this influence has been neglected, for one of the commonest resources of medical men in obscure cases is to lay all the mischief to the charge of "the stomach and bowels," and to mystify the patient by talking gravely about "indigestion" and "bile" as the origin of all his sufferings, where, if they were seriously taken to task, nothing would puzzle them more than to substantiate their assertions. But this is not the way in which Mr. Parker disposes of the subject. Aware from experience of the reality of the stomach's influence upon the course of disease, he first examines its healthy relations to other organs, and afterwards endeavours to trace their morbid sympathies by a constant reference to well marked and accurately observed cases; and having thus obtained definite and consistent facts, he next seeks to apply them to the better elucidation of the curative treatment.

In investigating this branch of the subject great caution is required not to confound affections which are really consentaneous results from a common cause, with those indisputably arising from the sympathy of one organ with another *previously* disordered. Thus, one of the most frequent complications of chronic gastritis is with affections of the liver, and this is generally accounted for by supposing the irritation to be propagated by "continuous sympathy," or otherwise from the stomach to the liver, and to excite vascular congestion and disturbance of function in it as well as in the stomach itself. In a great number of cases, we have no doubt that such is accordingly the true origin of hepatic disease; where, for example, the primary stomachic irritation arises in a person of active habits from intemperance or errors in diet, the first or direct action of which is upon the stomach. But there are numerous instances, as in people of sedentary professions exercised in a confined air, to which this explanation is inapplicable, and where the circulation in the liver, lungs, and heart becomes impeded, not so much from sympathy with the stomach as from being subject with it to the action of a common cause; and the mutual relation of the phenomena is merely their dependence on the same mode of life. This is not a mere theoretical distinction, for it bears directly on the success of practice. In both cases, a congested state of the stomach and liver is the real subject of treatment; but, in the one, the congestion depends entirely on local irritation inducing increased vascular action, first in the stomach and afterwards in the liver; and little more is required for its cure than to remove the stomachic irritation by appropriate diet and other means, after which the hepatic affection will subside from its cause being no longer in operation, and without any further change in the habits or mode of life of the patient; whereas, in the other, the congestion in both organs is the simultaneous effect of a cause acting equally upon both, and consequently the congestion of the liver being as much primary as that of the stomach itself, no form of treatment will prove available, *however appropriate to the state of the stomach*, so long as the impediment to the abdominal and hepatic circulation arising out of the constrained position and sedentary mode of life remains unremoved. It is for this very reason, indeed, that cases of indigestion with hepatic symptoms are met with among shoemakers, tailors, dress-makers, engravers, and clerks, which prove intractable under the usual remedies, even aided by a well-regulated diet, and yet are speedily cured by an accidental or intentional change to a more active profession. In all of the instances alluded to, the position of the body when at work is precisely such as directly to compress the epigastrium; to impede the free action of the diaphragm and abdominal muscles; and to prevent the due expansion of the lungs; or, in other words, to bring into active operation three most efficient causes of *hepatic*, *gastric*, *abdominal*, and *pulmonary* congestion: and if to these morbid influences we add that of errors of regimen, occasional intemperance or accidental exposure, we can have no difficulty in explaining why hepatic affections should often accompany indigestion; and why both, as branches from one root, should often spring up together and prove intractable so long as their real causes remain in activity. We have more than once seen young men, who were forced by circumstances to continue in a sedentary profession, observe every secondary precaution and take regular daily exercise, without being cured, and get well almost immediately on going into the country and leading a life of activity in the open air. The mere walk for an hour or two, which constitutes exercise in a town, is not sufficient to undo the mischief of *many* hours' constraint; and hence, though it palliates the symptoms, it fails to induce recovery.

Mr. Parker himself takes notice of this source of congestion, and quotes some excellent remarks from Portal on its influence in inducing diseases of the heart and general disturbance of the circulation; but, we think, he has scarcely attached to it so much importance as it really deserves, as an active and prevailing cause of bad health. At the same time, we admit that it would be easy to extract from the volume before us many proofs of the truth of the position we have just been enforcing, and there is one case in particular so perfectly opposite that we cannot refrain from quoting it. Mr. Parker was consulted by a gentleman "for what the latter considered mere prolonged and obstinate indigestion. On examining carefully into his state, I discovered," says Mr. Parker, "that he had extensive valvular disease of the heart, and that his *stomach disease resulted from an unusual quantity of blood retained in the mucous coat of the stomach and its veins, from a mechanical obstacle to its free return to the heart* caused by disease existing in that viscus." If indigestion and gastric congestion can thus be produced by a physical cause preventing the return of the venous blood, it cannot be denied that the same results *may* follow from any other kind of obstruction; and we have no doubt that hypertrophy of the liver and other abdominal diseases often take their origin in similar impediments of an accidental or organic kind. In the above case, of course, no cure could be effected, because no treatment could restore the heart to its normal state.

As additional illustrations, may be mentioned, that we have seen many instances in which we could distinctly trace what is called stomach cough, shortness of breathing, and palpitation, (which were previously ascribed to nervous *sympathy* with stomachic irritation,) to oppression of the pulmonary circulation from the same cause, acting at once upon the stomach, the lungs, and the heart, deranging the functions of all at the same time. When the lungs are inadequately expanded, the circulation through them is impeded, the vessels ramified on their mucous surfaces become loaded, the dilatation of the air-cells is thereby still further diminished, increased secretion takes place, and the absorbing power of the veins being impaired, cough is excited to relieve the sense of oppression thus induced. The increase of cough after eating, so often observed in such circumstances, seems to arise from the increased distension of the stomach acting as an additional impediment to the dilatation of the lungs.

To account for the irregularity in the action of the heart prevalent in indigestion, we have only to pursue the enquiry a step further. The free passage of the venous blood through the lungs being prevented, it follows that it cannot arrive in due quantity at the right side of the heart. As a necessary result from the want of its proper stimulus, the latter acts feebly, and sometimes does not contract till an unusual quantity of venous blood has accumulated in its cavities. It then beats violently three or four times in succession, and after a pause relapses into its former feebleness; and, by and by, the same phenomena are repeated, till, if the cause be long in operation, permanent dilatation of the right auricle and ventricle ensues. We have often seen—we might say we have experienced—what we now describe; and always in circumstances which left little doubt as to the real origin of the venous congestion, and demonstrated that the pulmonary symptoms and disturbance of circulation were not owing to sympathy with stomachic irritation, and that the whole phenomena were the direct and simultaneous consequences of a common cause. The influence of grief, anxiety, and other depressing passions, in inducing indigestion and other forms of disease, admits of a similar explanation. The natural activity of mind and body disappears for the time, and the respiration is slow and greatly diminished in extent. The lungs being imperfectly inflated do not afford free passage to the blood, and muscular action being impaired, the impulse to its circulation is also withdrawn. Hence arise nearly the same results as from physical constraint.

From a good deal of attention to the subject, we incline to think that this hepatic and abdominal and perhaps pulmonary congestion is more common and influential in the production of bad health, and especially of chronic diseases both of the stomach and lungs, than is generally believed; and those of our readers who are acquainted with the best German writers must be aware of the importance which they attach to abdominal engorgement in particular. It would lead us out of our present path to discuss the subject fully here; but the more narrowly it is investigated, the more convincing will the proofs become of the influence of causes of this nature, which in this country have till lately been almost overlooked, and without the removal of which recovery can scarcely be expected. We do not deny that affections of other organs are excited purely by sympathy with stomachic irritation. They frequently are so, and then the first step to their removal is its cure; but they occur chiefly in nervous and excitable constitutions, while the affections apparently sympathetic but really from a common cause are met with chiefly in persons of less nervous susceptibility. Often, however, the two are blended in one individual, and this is just another reason why the practitioner should, in every case, have his attention alive to all possible contingencies and not decide rashly where serious harm may be the result of error.

Our limits being completely exhausted, we must now take leave of Mr. Parker, although we have omitted a variety of extracts which we had marked for insertion, and left many points untouched which we were desirous to notice. The length of our review and even the nature of our strictures, are indisputable evidence of the interest we have felt in his work; and we can assure him that if we had believed it to be a merely ephemeral production, we should have been less anxious either to point out its defects or to offer suggestions for its future improvement. But like all books written for the purpose of adding to the mass of useful knowledge, and containing the results of careful and extensive observation digested by a well informed, truthful, and reflecting mind, the work before us is, we trust, destined to enjoy a far more vigorous and protracted existence than is likely to be the lot of most of its rival contemporaries.

---

